# Snakes and Ladders: The experience of being referred to and seen by Child and Adolescent Mental Health Services

**DOI:** 10.1016/j.ssmmh.2024.100343

**Published:** 2024-12

**Authors:** Kristina L. Newman, Kapil Sayal, Colleen Ewart, Alexandra Lang, Anupam Bhardwaj, Bernadka Dubicka, Tamsin Marshall, Louise Thomson

**Affiliations:** a Institute of Mental Health, Nottinghamshire Healthcare NHS Foundation Trust, Nottingham, UK; b Nottingham Trent University, Nottingham, UK; c University of Leicester, Leicester, UK; d Mental Health & Clinical Neurosciences, School of Medicine, University of Nottingham, Nottingham, UK; e STADIA Patient and Public Involvement co-lead, Institute of Mental Health, University of Nottingham, Nottingham, UK; f Faculty of Engineering, University of Nottingham, Nottingham, UK; g Cambridgeshire and Peterborough NHS Foundation Trust, Fulbourn, UK; h Division of Neuroscience and Experimental Psychology, University of Manchester, Manchester, UK; i Pennine Care NHS Foundation Trust, Ashton-under-Lyne, UK; j Berkshire Healthcare NHS Foundation Trust, Bracknell, UK

**Keywords:** Child and adolescent mental health services (CAMHS), Referral, Young people, Parents/carers, Clinicians, STADIA, Hybrid delivery

## Abstract

**Background:**

Referral processes in Child and Adolescent Mental Health Services (CAMHS) have been reported as stressful and inadequate by young people and parents/carers, who struggle during waiting periods for the referral outcome decision. The Covid19 pandemic was an unprecedented time of distress for young people, parents/carers, and healthcare staff, with increased mental health challenges and stretched staff having to adapt modes of care, thus exacerbating difficulties for CAMHS.

**Aim:**

This qualitative study aimed to capture the unique lived experiences of young people, parents/carers, and CAMHS staff during the referral process in the peak of the Covid19 pandemic.

**Methods:**

As part of the STADIA trial, between 2020 and 2022, 109 semi-structured interviews across 8 NHS sites were conducted with young people (aged 16–17), parents/carers, and NHS staff including clinicians, commissioners, managers, and researchers embedded in clinical services. Interviews were analysed using thematic analysis.

**Results:**

Three themes were elicited to express young people, staff, and parents/carer experiences of the referral process, CAMHS, and the impact of Covid19: 1) referral as a starting point; 2) changes to methods of appointment delivery and their effect on CAMHS experience; and 3) experiences and evaluation of services.

**Conclusion:**

Although CAMHS was seen as the pinnacle of mental health support, there was dissatisfaction with waiting times, limited communication, unclear referral processes, and limited clinical capacity and resources for young people, parent/carers, and staff. Covid19 forced CAMHS into adapting to a hybrid model of care, increasing accessibility for young people, parents/carers and staff and highlighting areas for improvement. Secure and consistent support and increases in staff resources are essential to address challenges with CAMHS delivery and improve the experiences of young people, parent/carers, and staff.

## Highlights

• In the UK, the referral process to Child and Adolescent Mental Health Services (CAMHS) can be distressing, often involving long waits and limited communication.
• Rejection of a referral may result in feeling lost and hopeless.
• Redirection to alternative services is perceived as disappointing if not handled sensitively.
• Following the pandemic, some young people, parents, and carers preferred the accessibility and flexibility of hybrid consultation.
• Clearer information and communication during long periods of waiting, or following referral rejection, are essential.

## 1 Introduction

In recent years, there has been an increase in the prevalence of mental health disorders among children and young people. The most recent wave of a national epidemiological survey in the United Kingdom (UK), highlighted that 20.3% of 8–16 year olds had a probable mental disorder in 2023 (Newlove-Delgado et al., 2023). This figure increased from 12.5% in 2017, 17.1% in 2020, 17.7% in 2021, and 19.0% in 2022. These statistics underscore the urgent need for enhancing appropriate and timely mental health support. Child and Adolescent Mental Health Services (CAMHS) in the UK are secondary healthcare services providing specialist multi-disciplinary mental health assessment and care to those under 18 years of age. CAMHS can be accessed through a referral from primary care services (General Practitioners, GPs), schools and self-referral in some cases. CAMHS have been characterised for many years by unclear and challenging referral processes and limited clinical capacity, leading to long waiting times (e.g. Rocks et al., 2020). CAMHS have been increasingly adopting a single point of access (SPA) model, aiming to streamline the referral process and allow for greater access through self or parent/carer referrals whilst maintaining the role of CAMHS as a gatekeeper of these services. Despite the SPA model goal of expediting access, recent investigations have shown evidence of more rejected referrals as capacity in services has not increased in line with demand (Rocks et al., 2020), and that referral rejection from CAMHS is more common for young people referred with emotional and behavioural difficulties (Smith et al., 2018).

In particular, the prevalence of emotional disorders has increased over the last two decades (Sadler et al., 2018). Emotional disorders (e.g. anxiety disorders, depression) are distressing for young people and their families, impacting on social relationships and overall quality of life (Sadler et al., 2018; Goodyer et al., 2017; Simmons et al., 2015) with risks of self-harm, and suicide (Goodyer et al., 2017; Orchard et al., 2017). Rates of referral acceptance into CAMHS vary depending on location, resulting in unequal access to and provision of quality mental health care, an issue highlighted in the 2022 NHS England recent review (NHS England, 2022).

With restrictions introduced during the Covid19 pandemic, the referral journey and intervention delivery changed to initially online and subsequently hybrid delivery at a time of great distress and uncertainty globally and added strain on NHS staff (Newman et al., 2022). In Ireland, referral rates initially decreased in the wake of the pandemic in March–August 2020, similar to rates in the UK (Chen et al., 2020). Referrals then increased sharply by 50% compared to 2018 and 2019, and 180% in November 2020, with double the number of outpatient appointments offered (McNicholas et al., 2021). In the UK, even amongst routine referrals to CAMHS, self-reported severity of difficulties and impairment increased in the periods following post-lockdown school re-openings (Sayal et al., 2022). This increased strain on services in- and post-pandemic is expected to have a lasting negative effect on waiting lists and resources (McNicholas et al., 2021). The impact of remote delivery of care on patient outcomes and experience is still not clear. One local NHS CAMHS (Bhardwaj et al., 2021) reported that remote delivery (68% of consultations by telephone and 31% by videocall) did not affect safeguarding or rapport with patients or the length of assessments, however, 28% of consultations were reported to have technical difficulties. Furthermore, we know little about the lived experiences of children, young people, and their parents/carers during this period, how this compared with their needs and expectations, and how it may have influenced outcomes.

Expectations in relation to healthcare are a key driver influencing the experience of and satisfaction with healthcare services (Lakin and Kane, 2022). Expectancy-disconfirmation theory (Oliver, 2010) has been widely applied to understand satisfaction with public services (Zhang et al., 2021) and proposes that satisfaction is largely determined by individuals comparing prior perception of a service with perception based on their actual experience (Oliver, 2010). Lakin and Kane (2022) propose that social structures and relations are also key to shaping healthcare-related expectations and satisfaction with services. During the COVID pandemic, the nature of these social structures and relations in CAMHS, and the broader contexts within which these services exist, were dramatically transformed. Most studies about child and adolescent mental health during and after the pandemic have focused on patterns of admissions or emergency referrals (e.g. Ferrando et al., 2020; Leeb et al., 2020; Nagiub and Hegde, 2021; Ougrin, 2020) or mental health symptoms and functioning (e.g. Barendse et al., 2023; Carey and Spratt, 2009; Mansfield et al., 2020; Sayal et al., 2022; Staite et al., 2022; Waite et al., 2021). Some studies within single CAMHS sites or teams have explored experiences of and satisfaction with remote provision of services during the pandemic identifying the acceptability of remote consultations (Agutu et al., 2021) but also the need for considering individual preference and access to space and hardware (Worsley et al., 2022). But how the changes influenced the development of expectations, and, in turn, the experience and satisfaction of young people and their parents/carers have not been extensively investigated across a range of sites.

This study aims to understand the experience of the CAMHS referral process during the peak of the Covid19 pandemic from the perspectives of young people, parents/carers, and CAMHS staff including clinicians, managers, and researchers across eight CAMHS sites in England as well as commissioners of CAMHS (i.e. the funders of services with responsibility for assessing population-level needs, planning and prioritising, purchasing and monitoring health services). Given the prevalence of emotional disorders (Sadler et al., 2018) and high referral rejections (Smith et al., 2018), this study focuses specifically on young people with emotional difficulties referred to CAMHS. This study also captures the shift in care delivery as CAMHS adapt to a more hybrid model.

## 2 Method

### 2.1 Design

This qualitative interview study was part of the STADIA trial assessing the effectiveness of the Development and Wellbeing Assessment (DAWBA) (Aebi et al., 2012) a standardised diagnostic assessment tool in CAMHS (Day et al., 2022). The interview study explored the use of the DAWBA and the wider experience of CAMHS referrals and service, and it is the latter which is described and reported in this paper. Semi-structured one-on-one interviews were conducted with three groups: staff working in or alongside CAMHS; young people aged 16–17 who had been referred to CAMHS with emotional difficulties (for example, anxiety, low mood etc; see Day et al., 2022) and parents/carers of children and young people who had been referred. The interview questions were designed with input from the trial Patient and Public Involvement (PPI) lead (CE), consultation with the trial PPI group and clinicians in the trial management group. Interviews took place between January 2020 and June 2022.

### 2.2 Participants

109 participants from 8 CAMHS sites took part in semi-structured interviews. Parents/carers and young people taking part in the STADIA trial (Day et al., 2022) were invited to participate in the qualitative study. Young people were recruited at age 16 or 17 years, but some were 18 at the time of the interview. Staff across 8 NHS sites were invited including service commissioners, managers, clinical staff, and researchers working on the study. Researchers involved in the STADIA trial were included among the participants as they were embedded within the services and directly interacted with parents/carers and young people as part of the trial recruitment and data collection process, and also with CAMHS clinicians and managers, so were able to give a unique perspective on the experience of accessing CAMHS. A purposive sampling strategy was used to achieve maximum variation (Suri, 2011) in the sample demographics for young people and parents/carers (see Table 1). Staff demographics were not collected to preserve anonymity. We aimed to interview equal numbers across sites but our final sample size was informed by the power of the information being gathered from the interviews within the three groups of participants suitable for the aim of the study, specificity of the sample, quality of dialogue, analysis strategy, and application to theory (Malterud et al., 2016). Interview data was richly detailed, with high quality of dialogue in all groups. This was suitable for the analysis strategy of thematic analysis to compare experiences and views across groups.

**Table 1:** Participant demographics.

	Young People	Parents/carers^a^	Staff
Number interviewed	15	38	56

Sites			
Nottinghamshire	3	6	
Cambridgeshire	5	5	
London	4	6	
Pennine Care	0	10	
Berkshire	2	9	
Gloucestershire	0	2	
Rotherham and Doncaster	0	0	
Surrey and Borders	1	0	
Gender			
Male	2	6	–
Female	13	32	–
Other	0	0	–

Ethnicity			
White	11	34	–
Indian	0	2	–
Pakistani	1	0	–
Bangladeshi	1	0	–
Mixed ethnicity	0	2	–
Other	2	0	–

Age of index child			
5-10	–	12	–
11-15	–	19	–
16-17	15	7	–

Prior experience of CAMHS			
Yes	4	12	–
No	7	23	–
Unknown	4?	3	
Staff role			
Clinician	–	–	21
Team/Service Manager	–	–	14
Commissioner	–	–	6
Embedded STADIA Researcher	–	–	15

a 2 grandmothers are included in the carers.

### 2.3 Procedure

Potential participants who had consented to be contacted were sent further information about the interview study. If potential participants agreed to continue, an information sheet and consent form were provided. Interviews were arranged based on participant preference in relation to time and location (where COVID restrictions allowed) and method of communication (online or telephone), young people and parents/carers were offered a £10 voucher as a reimbursement for their time and contribution on completion of their interview.

For staff participants, information about the qualitative study was shared through the STADIA trial site Principal Investigators (PIs) via email and word-of-mouth. Interested staff contacted the researcher directly, and role-appropriate information sheets and consent forms provided. Demographic details of staff participants were not collected to protect their anonymity.

Interviews were largely conducted online (via Microsoft Teams) or by telephone, except for one face-to-face interview before the pandemic restrictions came into place. Due to covid-19 restrictions limiting access to equipment such as printers and postal services needed for written consent forms, some participants gave oral consent which was audio recorded before the interview. The interviews were audio recorded via an encrypted Dictaphone. Participants were then debriefed. Encrypted data was transcribed prior to analysis.

### 2.4 Analysis

Transcribed data were coded and analysed in NVivo, using the six steps of reflexive thematic analysis (Braun and Clarke, 2006; Braun and Clarke, 2020.); familiarisation by reading and rereading transcripts, coding of transcripts, initial theme generation, theme development and review, refining and defining, and writing up. An open coding approach was conducted by one researcher (KN), with initial codes being subsequently merged into larger categories of codes, and the subsequent sub-themes and themes discussed by two researchers (KN and LT). Extracts from a selection of anonymised transcripts were also coded by the trial PI, PPI lead, a clinician, and qualitative lead. This procedure allowed the research team to discuss the codes that had been applied to the data and why, check alignment and confirm the approach being taken by the primary coder (KN). This step also ensured that clinical, young people, and parent/carer perspectives were considered in the coding, interpretation of data and generation of themes. The trial management group including the site leads and PPI lead provided feedback on the initial themes, and their feedback used in theme development.

## 3 Results

Three themes were developed from the data to express participants’ experiences of the referral process, CAMHS, and the impact of covid19 pandemic: 1) referral as a starting point; 2) changes to methods of appointment delivery and their effect on CAMHS experience; and 3) experiences and evaluation of services: Frustration and misaligned expectations. Full theme information can be found in Table 2.

**Table 2:** Themes and descriptions.

Main theme	Sub-theme	Description
Referral as a starting point	Accepted referral and hope for change	Participants felt being accepted into CAMHS was the beginning of getting true help and seeing improvement.
	The disappointment of rejection and the revolving door of re-referral	Rejected referrals could cause despair, disappointment and feeling lost. Being passed on to different services was met with disappointment in parents/carers and young people.
	Waiting times and managing crisis	Long waiting times with limited to no communication was difficult for parents/carers and young people, who had to continue to manage the difficulties
Changes to methods of appointment delivery and their effect on CAMHS experience	The virtual learning curve of video appointments	Adjusting to video appointments was challenging for some clinicians but provided flexibility for staff and young people.
	Improved access but reduced privacy with remote appointments	Some young people were more comfortable not being seen but clinicians and parents/carers felt it was harder to engage the young person. Limited privacy in virtual appointments was difficult for some young people who did not want to discuss specific issues with their family.
	In person interaction perceived as best care	Face-to-face was felt preferable by all groups due to body language, having less distractions, and allowing 16–17-year-olds privacy from family.
	Looking to the future of CAMHS	Following learning from the pandemic, CAMHS delivery is expected to be more hybrid
Experiences and evaluation of services: Frustration and misaligned expectations	Staff needs: Supporting clinical teams to manage workload	Staff highlighted workloads being unsustainable with limited resources leading to increased waiting times.
	Areas to improve for better CAMHS experience	Young people and parents/carers experienced good and poor practice but highlighted better communication and reduced wait times as essential.

### 3.1 Theme: referral as a starting point

The acceptance or rejection of a referral by CAMHS was a key event defining the experience of young people, parents and carers. The long waiting times prior to that decision were identified as an uncertain time, leading to a range of emotions during that period of waiting and once the referral decision was communicated. Some young people and parents/carers waited more than a year to navigate their referral outcome and receive an initial assessment. This process is more protracted in the case of re-referrals, with some parents/carers waiting many years for support. Where resources allow, many families felt forced to pay for private care.

*“Appalled, let down, distraught. […] I was really horrified that we had to end up paying so much money to get that treatment” –* Mother 1

“*My experience, okay, it's been a dreadful experience because we've been trying to get support from CAMHS since early 2019 [3 years] and we've just got that support and it came far, far too late and has caused my daughter more harm and we've spent thousands now on private treatment in order to get some help because we couldn't wait any longer.*” – Mother 1

Parents/carers described CAMHS as ‘a ray of hope’ and the only way to access support, meaning that a referral acceptance was a high-stakes decision and often very emotionally charged, which was further exacerbated by long waiting times prior to the decision. Where referrals were accepted, the acceptance felt like a starting point and confirmation that difficulties were present, which was validating for young people and parents/carers.

*“Happy, yes, just because we’d been waiting, thinking ‘did he need help, did he not?’ so to know that someone would help him finally, that takes a bit of weight off my shoulders to be honest.”* – Mother 2

Having a referral acceptance was perceived as providing opportunity to try to receive a diagnosis, which was seen as the gateway to access the best clinical support and as well as opening doors to support in education and work settings. Where referrals were rejected or another service was recommended, this outcome was often met with anger and despair from young people and parents/carers, especially after long waiting periods. Many participants with this experience were tearful in interviews and felt they had been abandoned by services and did not know what to do.

“*It [rejected referral] kind of made me feel like I wasn't valid, do you know what I mean? That like my problems weren't good enough*.” – Young person 1

“*If we have to go back to the doctor, I don’t know, because the only place he can refer us is back to CAMHS, and CAMHS will have already gone ‘nah’ at that point. So, you know, I don’t know what we’d do next, I don’t know.” –* Father 1

Staff were also aware that waiting times were long and felt a sense of guilt, especially in the case of rejected referrals where patients may be turned away from CAMHS only to begin the process again.

“*I feel very sorry for the person who’s waited a heck of a long time for an assessment, …. it’s frustrating for them and a waste of our time.”* – Clinician 1

Services were under a lot of strain during the pandemic, with increases in referrals and reduced workforce due to restrictions, isolation, and illness, making it difficult to meet demand. The complex service landscape and chosen commissioned services were difficult to manage for both staff, and young people and parents/carers. Some who had been “rejected” immediately re-referred, either themselves or through a GP or education settings, to try again to get support. CAMHS staff acknowledged the frustration that young people and parents/carers felt but rationalised referral decisions made in relation to the greater suitability of alternative services and limitations in clinical capacity and commissioned resources to see every referral. Commissioners also acknowledged that frustration over rejected referrals was a key issue and that the lack of communication around rejection is also difficult for referrers, especially GPs.

*“I think it can be really devastating for families because you have got to recognise this isn't the first door they come to for early support, …, actually I think that is really frustrating but also disorientating for families because what they are saying is one set of professionals have said my child needs more support but the service …… is not willing to offer it to them so I think it is incredibly tricky.” –* Commissioner 1

The frequency of a rejected referral reinforces the need to have clear and accessible information about referral criteria and alternative sources of support including on-going communication during waiting times. One Commissioner referred to the need for more awareness amongst referrers of the right door and the wrong door for different levels of need to be met.

“I think from professionals they get quite frustrated when they've made a referral and it is rejected. I think having the wrong door policy is something that we should all kind of work towards to ensure if perhaps they haven't come to the right group, they are supported to get to the best place to meet their needs.” – Commissioner 2

Staff discussed how eligibility of referrals can be a challenge, as referrers and those referred have expectations of being accepted into CAMHS and may not be aware of the availability or appropriateness of alternative services. Signposting or re-directing to other services is an important aspect of CAMHS as funding limitations mean that they do not have the capacity to accept every referral. While some young people and parents/carers may perceive signposting to other services as rejection, staff described signposting as a different route to accessing the most appropriate types of support. However, timely and sensitive signposting was important and there were concerns of additional waiting lists for the external services once redirected. Where referrals had been redirected, young people and parents/carers were often disappointed and felt they were receiving a sub-optimal service. In contrast, others were grateful to have something else to try rather than being left lost as to where to try next. Managing expectations about the possibility and likelihood of a referral being redirected or signposted to a different and more appropriate service could help to manage these perceptions and experiences.

Waiting for a referral outcome decision was difficult for parents/carers and young people. Although it was generally understood that waiting times reflected limited funding and resources, participants felt uncertain how best to manage things in the interim while waiting to hear if their referral was accepted and were dissatisfied with the process overall. Clinical staff sympathised with the confusion and frustration expressed by young people and parents, understanding that they want help and that the process can be long and challenging. Clinicians were also concerned that families did not fully understand what CAMHS could and could not help with, and that rejected referrals could potentially lead to a revolving door involving re-referrals and further rejected referrals, increasing distress in the parents/carers and young people as they repeatedly attempt and fail to access support that they feel they need.

### 3.2 Theme: changes to methods of appointment delivery and their effect on CAMHS experience

With the limitations of the Covid19 pandemic and recommendations for isolation and social distancing, mental health services were forced to adapt and move the assessment process online or via telephone. Initially, referral rates dropped (McNicholas et al., 2021; Chen et al., 2020) as many school or GP referrals stopped due to school closures and many other services closing. This was then met by a sharp increase in referrals, echoing the report on Irish referrals from McNicholas et al. (2021), as the pandemic continued, symptoms worsened, and mental health declined following long periods of social isolation.

Some participants preferred the flexibility and accessibility of online appointments. During legally enforced pandemic-related full and partial lockdowns, periods when schools, colleges and most workplaces were closed, parents/carers who were required to work from home had more availability for online appointments and children and young people were easier to reach.

*“I think for [child] he quite likes technology, and he tends to do a fair bit online anyway so for him I think it was easier than meeting somebody face to face, it’s a bit more daunting.” –* Father 2

During other periods, the option of online and telephone appointments allowed more flexibility and accessibility around school and work hours for young people and parents/carers and removed transport and physical distance from the NHS service as a barrier. For staff, remotely-delivered sessions also saved time by not having to travel between sites or commute, allowing more time to focus on young people. Staff did identify that this blurred the lines between work life balance, often reporting working longer hours and struggling to switch off. During the school closure periods those staff with children also found it difficult to juggle home schooling and childcare, though those with younger children (with no home-school pressure) reported a better work-life balance.

However, it was felt by parents/carers and staff that some young people did not engage as well with mental health professionals via a screen.

“*Because whilst he’d become, or had to adjust, with engaging with people online, he would prefer not to. Particularly when it’s the first-time meeting somebody and there’s a- meeting a stranger over the- in 2D, on the screen, would have been more daunting than meeting them in person.”* – Mother 3

Furthermore, some young people highlighted that they felt uncomfortable with their parents/carers being present or nearby in appointments and expressed a desire for privacy which was difficult to achieve remotely. This was more apparent in young people from minority ethnicities, where family members were not aware of them seeking mental health support.

*“I wanted to be in a different environment, I would have felt much more comfortable outside of the house, and I remember being on the [psychological intervention] course and I remember I felt that I had to be quieter and I just didn’t want anyone to hear me, I just wanted to […] and I wanted to without having to worry, so yeah face-to-face would have been a lot better.” –* Young person 2

*“I don’t like online appointments […] because, ‘cause my family’s horrible, they listen, they get all up in my face and they don’t like me, they just … they just like to make fun of me, I guess so, I don’t like being hurt if I’m honest, so as much time out of the house as I could get.” –* Young person 3

There was considerable individual variation in the preferences for different methods and platforms for delivering remote appointments. Telephone appointments were generally less popular with parents/carers and staff as they missed important body language cues, and it was more difficult to tell if the young person was engaged in the call. But some young people preferred telephone appointments because they were self-conscious, and it allowed them to not be seen by the clinician. Phone calls were also more accessible for some as they did not require an internet connection, and older parents/carers who struggled with technology appreciated having phone calls as an option. Face-to-face appointments were believed to be the most effective by clinicians and parents/carers, and some young people also preferred the opportunity to be seen in a private room outside the family home for sensitive conversations about mental health. Clinicians felt that certain facial and body language cues reveal insight into how the young person was feeling could be helpful in understanding the patient more effectively.

“*We have done local engagement with children and young people, and they have said they want to go forward and have mixed access, they definitely don’t want it only being online and over the phone, but it has worked for some.*” - Commissioner 2

Coming out of lockdown periods, online appointments remained popular as they offered flexibility around school and work schedules of young people and their parents/carers, with many routine CAMHS closed outside traditional work hours. However, young people, parents/carers and staff all identified that the option of face-to-face appointments was likely to remain the most preferable and should continue to be offered in the future.

*“I think it [COVID] forced the [service] to speed up on some of the IT systems which I don't think would have happened had they not been forced to; I think probably we wouldn't have gone to video appointments for several more years*.*”* – Clinician 2

“*We’re more adaptable in our working with our computers at home and using Teams. I don’t think we used Teams before Covid came, yes, it will make us more efficient, can record stuff. I run groups with parents, and I think it’s made it that we’ve had more fathers join the groups, it’s made it more accessible for some families and appointment times and things, people can do them in their lunch hour so yes, I think it's been a good thing in bringing CAMHS more up to date.”* – Clinician 1

Staff reflected on the lessons learned from hybrid and online ways of working during the pandemic and what this might mean for the future. Staff felt the electronic systems in the NHS were outdated and challenging to use and that the pandemic had forced CAMHS to catch up and move into the digital age. Staff reflected that the use of video appointments was likely to continue, and they felt positive about a hybrid model as some had a strong preference for face-to-face clinical delivery, whereas others enjoyed the flexibility of online or remote. The option for holding clinical team meetings virtually was also perceived useful as this reduced time and travel constraints. Young people and parents/carers were also positive about hybrid delivery, with the understanding that face-to-face appointments would still be an option depending on the young person's individual needs and preferred method of communication.

### 3.3 Theme: experiences and evaluation of services: frustration and misaligned expectations

For those whose referrals into CAMHS were accepted, many were unhappy with their experience of the service following long waits and perceived appropriateness of assessment and support offered. They described feeling stuck in an endless loop of care that never quite reached the type of service or treatment that they hoped for, referring to the children's board game Snakes and Ladders (also known as Chutes and Ladders and Moksha Patam).

“It's like snakes and ladders, you should be able to go up the ladder straight to the psychiatrist door but you're not, you're having to go through all this pathetic useless stuff that everyone knows doesn't work because we've been […] it for years and years […], it is a queue, it's just a queue that's all it is” Mother 1

Some felt they were not listened to and had concerns and contextual trauma dismissed in the assessment, leading to disappointment in the service received after waiting a long time to be seen. Assessments sometimes felt rushed or uninformed and parents/carers were concerned screening questionnaires were not looked at by clinicians, leaving them and young people frustrated and disappointed.

“*The main issue with work is that people have been waiting longer, so because they’ve been waiting longer there’s more pressure to treat quicker or people are already angry about how long they’ve been waiting so I think people coming with more expectation or worse symptoms as well […] we want them to have the therapy alongside the medication but they haven’t’ always had that because of the waiting times*.” – Clinician 3

Some parents/carers were redirected to parenting courses they had already completed, and some young people were redirected to services offering relaxation rather than direct support, leading to frustration as these were perceived as inappropriate. Many had expected and hoped to receive a diagnosis, which would in turn facilitate accessing medication treatments which they believed was needed and would directly improve the condition.

*He just said to me "Oh well, what do you want?". He looked at my daughter and was kind of puzzled and thought there was nothing to deal with. Well, I said to him "Look, we are still waiting for an official diagnosis in the meantime". Well, then he said straight away "I don't deal with diagnosis". –* Mother 4

There was also a sense of time running out for some young people approaching the age of 18 years, or 16 in some services, who were concerned they will not be seen by CAMHS while they are still within eligible age range, and uncertainty around the process for transfer to Adult Mental Health Services (AMHS). However, once transferred into AMHS, young people felt the service was more organised and accessible than CAMHS with diagnosis and treatment options being accessed much quicker. Private mental health support was viewed relatively positively by parents/carers in providing access to diagnosis and support; however, the expense was a big barrier.

Overall, clinicians and commissioners identified that services did not have the capacity to deliver the care young people and parents/carers were looking for as the demand was so high and there are not enough resources in terms of time and staffing to meet these demands. This lack of capacity led to longer waiting times, frustration from young people and parents/carers and expectations not being met.

“*There has been a massive rise in demand for services which is really challenging because there is not enough workforce out there to be able to deliver services to meet all of that demand.” –* Commissioner 3

Nonetheless, despite these challenges CAMHS was perceived as the peak of mental health care and the desired source of support by young people and parents/carers.

## 4 Discussion

This large national qualitative study identified important evidence about the referral process in CAMHS from the perspectives of young people, parents/carers and staff, and the Covid19 pandemic as a driver of change for how CAMHS services are delivered and experienced;

The findings highlight the importance of young people and their parents/carers' expectations about referrals and their outcomes in shaping their experiences and perceptions of the referral process. At the individual-level, there appears to be belief-outcome expectation (Lakin and Kane, 2022) amongst many young people and parents/carers that it is a referral into CAMHS that is the key to addressing their mental health needs. The hope and expectation is that their referral will be accepted by CAMHS and then, in turn, lead to assessment with a specialist (i.e. someone who has expertise in the area) followed by a clear diagnosis and treatment plan. However, it is clear from our findings that this is not the reality for most referrals and this misalignment furthers feelings of dissatisfaction, frustration and despair amongst young people and their parents/carers. There is opportunity for patients and referrers to develop improved mental models (Wickens et al., 2013 p. 236-8) about CAMHS referral processes and decisions. Mental models are the mental structures that reflect a user's understanding and expectations of a system or the ‘world’, inclusive of decision-making processes and anticipated responses (Wilson and Rutherford, 1989; Holtrop et al., 2021). Whilst prior research has shown that incongruence between patients' treatment expectations and the actual psychiatric care they receive can lead to negative outcomes (e.g. Koekkoek et al., 2010; Noble et al., 2001), our findings suggest that the experience during the referral process and waiting times are also important. Some of the negative experiences and perceptions described by our participants could be avoided by improving service users' and referrers' understanding of the CAMHS referral process and possible outcomes as well as making the decision-making process more transparent and communicating referral decision and outcomes more clearly. This can manifest in better alignment of how the CAMHS system works with the likely experience and outcomes, through more effective communication and management of realistic expectations. Subsequent benefit may then be experienced if potential frustration can be reduced, e.g in a scenario with a referral rejection, when families are signposted to a different service and realise that alternative services might be more appropriate to address their needs. This, in turn, may prevent revolving doors of re-referrals and deteriorating mental health. Feedback explaining the reason for a referral rejection was thought to be important. Where young people report severe symptoms, it can be distressing and confusing as to why they are not directly accepted by CAMHS, or why clinicians are unaware of details from the referral. Clinicians and commissioners reflected that GPs, as the initial referral source, may also be concerned if there is not clear feedback about why the referral is rejected and what alternative steps are recommended. Transparency of decision making and methods of Signposting or Redirection of referrals with recommendations for another service, and how this is communicated to GPs, schools, parents/carers and young people, is important as this must be handled sensitively to prevent it being perceived as invalidating and as a rejection. Offering a clear rationale and the expected outcomes may help to put young people and parents/carers at ease, while those who have already been through the recommended service or do not wish to try this should be able to discuss alternative options to allow for autonomy rather than experience a sense of dismissal or failure. This may address that many families felt forced to pay for private care to access support quicker, however most families do not have this route as an option. This, in turn, reinforces health-related inequalities.

Further implications arising included consideration of referrals and whether relevant information was provided to make decisions on suitability for acceptance into CAMHS. Following this, the receipt and handling of referrals once received by CAMHS is in clear need of improvement to support young people, parents/carers, and staff. The outcome of referral decisions and the next steps required are also in need for improvement, as communication issues and distress at rejected referrals are not addressed, leaving some families in continued or higher levels of crisis. Referrals are usually made at the point where difficulties are their worst. For accepted referrals, the long wait periods are often without support and symptom severity may increase which adds to clinician and young person burden. When reaching the stage of assessment, some young people and parents/carers report dissatisfaction with care received and feel they are not listened to or taken seriously. Patient-centred and mutually agreed care should be allowed where possible to give young people and parents/carers autonomy of their care and an opportunity to be listened to, respected, ensure understanding and build rapport with healthcare professionals.

The uncertainty and distress around waiting times was clearly identified as an issue that needs to be immediately addressed. When looking at interventions to reduce waiting times, a systematic review (Thomas et al., 2021) identified various strategies utilised before Covid19 and at lower prevalence rates. For example, telephone triage allowed clinicians to identify patients in crisis, and for recommendation to alternative services (Hardy et al., 2011; Melathopolous and Cawthorpe, 2019; Jones et al., 2013). Obtaining sufficient information before the referral outcome decision is made, to streamline the referral process may address Frith's (2017) finding that referrals are commonly rejected due to lack of information. A fortnightly triage in one service cleared all new triage cases at each session (Evans, 2014). Patient partnership approaches resulted in shorter wait times for first appointments (Clark et al., 2018; Naughton et al., 2015, 2018), with more first appointments offered (Wilson et al., 2015), and positive service outcomes for users (Robotham et al., 2010). Walk-in clinics also reduced or cleared wait lists (Barwick et al., 2013; Neufeld et al., 2012; Stalker et al., 2016). In multi-disciplinary approaches, employing healthcare professionals in primary care also reported a reduction in waiting times (Cordeiro et al., 2015; Haggarty et al., 2012). Even if wait times can't be reduced, it might be possible to offer access to waiting-list interventions during the wait period prior to the first appointment Valentine et al. (2023).

However, while promising results, all these interventions were conducted prior to Covid19 where demand was already increasing substantially (Newlove-Delgado et al., 2022), and all require financial and staff resources to implement long-term while the service is already struggling. Significant funding, recruitment of additional staff, and training is required to implement any of these strategies on a large scale, which will be difficult with over 1500 WTE vacancies in CAMHS reported in 2021 (NHS Benchmarking Network, 2021), reflecting a 15% increase in FTE doctors in child and adolescent psychiatry in comparison with a 327% demand increase between April 2016 and April 2023 (British Medical Association, 2023). Future interventions should prioritise easing staff burden, increasing communication and information for young people and parents/carers, and prioritising choice and flexibility in care delivery.

Staff reflected that the use of video appointments was likely to continue, and they felt positive about a hybrid model with options for face-to-face clinical delivery or working online or remotely. With potential benefits of remote delivery including improved accessibility, reduced travel and no impact on safeguarding, rapport, or session duration (Bhardwaj et al., 2021), standardised remote delivery options would be beneficial. Young people and parents/carers were also positive about hybrid delivery, with the understanding that face-to-face appointments would still be an option depending on the young person's individual needs and preferred method of communication. However, young people, parents/carers and staff all identified that the option of face-to-face appointments was likely to remain the most preferable and should continue to be offered as an option in the future where possible. There are considerable implications for service delivery and management to enable the potential benefits of online appointments whilst minimising the risks and also supporting flexibility and choice both for service users and staff. As well as individual preferences, there are other factors to consider such as team culture, clinical needs (e.g. the need for observations for some diagnoses) (Bhardwaj et al., 2021). Other studies have reported difficulties clinicians may have in identifying non-verbal cues, building rapport and picking up risks when working remotely (Bentham et al., 2021; CQC, 2022; Worsley et al., 2022) which alters the therapeutic experience (Shaw et al., 2021). Worsley et al. (2022) also reported benefits to young people of being in an in-person therapeutic space which is away from the home environment which can be a place of trauma or of perceived comfort and safety. Ensuring that young people, parents/carers and staff have the appropriate skills, equipment and space for conducting online appointments needs careful consideration. There are risks that services become less accessible and inclusive for those without digital access and skills (Worsley et al., 2022). Staff, parents/carers, and young people's preferences and efficiency of services should be balanced, with flexibility of choice to enable a blended model of service provision for optimum care.

This multi-site study identifies referral experiences in young people, parents/carers, and a variety of staff including clinicians and commissioners in a large sample of interviews. The diversity in perspectives, paired with the catalyst of Covid19, forcing services to capacity limits and driving change to existing processes, provides a powerful narrative of issues in the referral and assessment process and the need for change. In future research, consideration of these views and the wider CAMHS care models would be beneficial in developing interventions and policies and inform commissioning. Limitations include a lack of diversity, despite attempts through purposive sampling, participants were majority white, female, and had negative experiences. Future research should also aim to capture minority voices to overcome barriers to the inclusion and design of services for those of whom cultural stigma can be a barrier to engaging with mental health services. These enquiries should also assess additional barriers to care access for those from different socioeconomic backgrounds, including barriers to reporting mental health concerns, engaging with services, and recruitment to studies, and how to best offer support to young people referred without the knowledge and support of their family.

## 5 Conclusion

CAMHS has been under enormous pressure over recent years with clinical capacity more stretched than ever before in the context of the Covid19 pandemic, and significant workforce gaps. Parents/carers and young people are dissatisfied with long waiting times, lack of communication while waiting, rejected referrals without clear options for next steps, and dissatisfaction with assessments and care provided by CAMHS. Timely and sensitive signposting is important and whilst hybrid clinical support offers flexibility and increased accessibility, care must be taken to best meet individual needs and preferences. Care must also be taken to manage referral expectations and interventions must be put in place to reduce clinical burden in CAMHS and reduce waiting times, improve accessible information, and clear communication about other services.

## Funding

This study was funded as a result of a commissioned call by the 10.13039/501100000272National Institute for Health Research (10.13039/501100000272NIHR) Health Technology Assessment programme (Grant Reference Number 16/96/09).

## Disclaimer

The views expressed are those of the authors and not necessarily those of the NIHR or the Department of Health and Social Care. The funder will have no role in the collection, management, analysis, and interpretation of data; writing of the report; and the decision to submit the report for publication.

## CRediT authorship contribution statement

**Kristina L. Newman:** Writing – review & editing, Writing – original draft, Project administration, Methodology, Investigation, Formal analysis, Data curation, Conceptualization. **Kapil Sayal:** Writing – review & editing, Resources, Project administration, Funding acquisition, Data curation, Conceptualization. **Colleen Ewart:** Writing – review & editing. **Alexandra Lang:** Writing – review & editing. **Anupam Bhardwaj:** Writing – review & editing, Data curation. **Bernadka Dubicka:** Writing – review & editing, Data curation. **Tamsin Marshall:** Writing – review & editing, Data curation. **Louise Thomson:** Writing – review & editing, Supervision, Resources, Methodology, Formal analysis, Data curation, Conceptualization.

## Declaration of competing interest

The authors declare that they have no known competing financial interests or personal relationships that could have appeared to influence the work reported in this paper.
