# Supplementary material for: Snakes and Ladders: The experience of being referred to and seen by Child and Adolescent Mental Health Services
Source: SSM Ment Health. 2024 Dec;6:100343. doi: 10.1016/j.ssmmh.2024.100343 (PMC11635093; doi:10.1016/j.ssmmh.2024.100343)
Supplement: Multimedia component 1 [file mmc1.docx]

**Supplementary Material:**

**Interview Questions**

**Young People/Parents Carers**

- What has been your previous experience of CAMHS?
- Have you been referred before? What was the outcome of this?
- Could anything have been done better?
- How do you feel about waiting times?
- If you are/were not accepted into CAMHS, how would/did this make you feel?
- What effect has Covid19 had on you?
  - Do you think it has had an impact on your referral?
  - Online vs face to face

**CAMHS Clinical Staff**

- What normally happens after a patient is referred?
  - How do you conduct an assessment?
  - What are the aims of your assessment?
  - Do you routinely discuss your assessment / formulation / provisional diagnosis with a colleague? Is this covered in clinical supervision?
  - What happens if a patient is not referred? How does this make you feel?
  - How has this been different during Covid19?
    - Online vs face to face
    - Referral numbers
    - What effect, if any, do you think Covid19 has had on this study?
    - Online vs face to face
    - Waiting times
    - Quality of care

**CAMHS Triage Staff**

- What normally happens after a patient is referred?
- What happens if a patient is not referred? How does this make you feel?
- How does this differ from the intervention in this research study, in which the DAWBA tool is used?
- How has this been different during Covid19?
- Online vs face to face
- Referral numbers
- Has your service been outsourcing to private providers more since covid19 began?

**CAMHS Managers and Service Commissioners**

- What do you hope to learn from this particular research study? How might you implement this learning into practice in this service/Trust? How might it inform or impact on commissioning?
  - Number of referrals received;
  - Ensuring most appropriate referrals are accepted.
  - Effects on families or referrers when a referral is turned down.
- What do you see as the potential benefits of your service/Trust being involved in a research study like this?

**Research Staff**

- What effect, if any, do you think Covid19 has had on this study?

N.B. In the interests of brevity, additional interview questions on topics that weren’t the focus of this paper are omitted from this Supplementary Material. These additional questions related to:

For young people and parent/carers

- participants decisions to take part in the study
- participants experience of being in the study
- participants experience of using the DAWBA
- participants experience of being part of the STADIA trial

For CAMHS clinical and triage staff, managers and commissioners

- how DAWBA has been used and implemented in clinical practice
- approaches to diagnosis

For research staff

- retention of participants in the study
- feedback on the DAWBA
- how DAWBA has been used and implemented in clinical practice
- approaches to diagnosis within the service
